# A260 ADVANCING INFLAMMATORY BOWEL DISEASE DIAGNOSIS THROUGH STOOL PROTEOMIC SIGNATURES OBTAINED VIA DIA-MASS SPECTROMETRY AND MACHINE LEARNING

**DOI:** 10.1093/jcag/gwad061.260

**Published:** 2024-02-14

**Authors:** E Shajari, D Gagné, M Malick, M Brunet, M Delisle, F Boisvert, J Beaulieu

**Affiliations:** Universite de Sherbrooke Faculte de Medecine et des Sciences de la Sante, Sherbrooke, QC, Canada; Universite de Sherbrooke Faculte de Medecine et des Sciences de la Sante, Sherbrooke, QC, Canada; Universite de Sherbrooke Faculte de Medecine et des Sciences de la Sante, Sherbrooke, QC, Canada; Universite de Sherbrooke Faculte de Medecine et des Sciences de la Sante, Sherbrooke, QC, Canada; Universite de Sherbrooke Faculte de Medecine et des Sciences de la Sante, Sherbrooke, QC, Canada; Universite de Sherbrooke Faculte de Medecine et des Sciences de la Sante, Sherbrooke, QC, Canada; Universite de Sherbrooke Faculte de Medecine et des Sciences de la Sante, Sherbrooke, QC, Canada

## Abstract

**Background:**

Resembling symptoms between flare-up Inflammatory bowel disease (IBD) and other gastrointestinal diseases and conditions, make the initial diagnosis and accurate treatment of this disease challenging. Currently, the most common clinical methods used for diagnosing and monitoring IBD are colonoscopy and biopsy, which are invasive and uncomfortable procedures, and the fecal calprotectin test, which is not sufficiently accurate. Therefore, it is necessary to develop an alternative method.

**Aims:**

In this study, our aim was to develop a robust predictive model for a noninvasive and accurate test to distinguish active IBD from non-IBD controls.

**Methods:**

A total of 120 samples were collected, with 78 samples separated for retrospective analysis and model training, while 42 samples were set aside for prospective validation. We employed SWATH mass spectrometry for the identification and quantification of the stool proteome. The obtained data underwent multiple stages, including data preprocessing, feature selection, model training, and performance evaluation. We optimized data processing procedures through advanced bioinformatics, selecting an appropriate pipeline that included data normalization, batch effect correction, and missing value imputation. Subsequently, we assessed various machine learning algorithms to determine the most effective classifier for predicting IBD based on the selected proteins.

**Results:**

After data preprocessing, we identified 48 differentially abundant proteins (DAPs). To eliminate redundant proteins, we employed Correlation-based Feature Selection (CFS), resulting in selecting 7 proteins. To identify the most suitable predictive model for our dataset, we assessed five popular machine learning methods: Support Vector Machines (SVM), Random Forests, Logistic Regression, k-nearest neighbors (KNN), and Naive Bayes. Among these, SVM exhibited the highest performance. Finally, we evaluated the model's performance by applying the selected algorithm to 42 prospective blind samples. The results revealed a sensitivity of 96% and a specificity of 76%, highlighting its strength and effectiveness.

**Conclusions:**

In conclusion, this study offers a proof of concept for the application of SWATH for precise IBD diagnosis using stool proteomics and showcases the effectiveness of our data processing and machine learning approach. Additionally, it highlights the potential of this method for classifying Crohn's Disease (CD) vs. Ulcerative Colitis (UC) and distinguishing active IBD from remission.

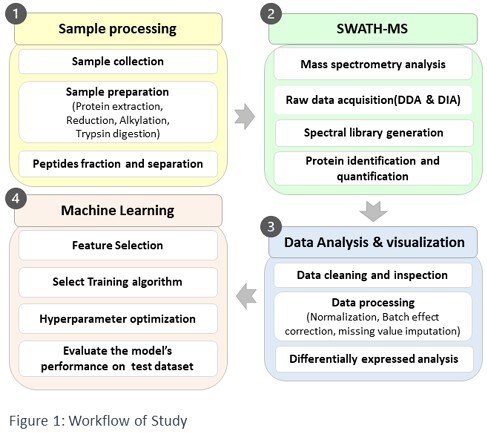

Workflow of study

**Funding Agencies:**

CIHRCrohn's and Colitis Canada , Studentship from CRMUS,

